# Understanding service users and other stakeholders’ engagement in maternal and newborn health services research: A systematic review of evidence from low- and middle-income countries

**DOI:** 10.1371/journal.pone.0309888

**Published:** 2024-11-27

**Authors:** Devendra Raj Singh, Rajeeb Kumar Sah, Bibha Simkhada, Zoe Darwin

**Affiliations:** School of Human and Health Sciences, University of Huddersfield, Huddersfield, United Kingdom; Kandahar University, Faculty of Medicine, AFGHANISTAN

## Abstract

**Background:**

Stakeholder engagement is widely considered democratic, transparent, and essential in the shared decision-making process for improving health services. However, the integrated evidence of stakeholders’ engagement activities in maternal and newborn health (MNH) services in the context of low- and middle-income countries (LMICs) is lacking. Therefore, this review aims to generate synthesised evidence of different practices for stakeholder engagements, characteristics of stakeholder engagements and outcomes of stakeholder engagements in improving the MNH services uptake and delivery.

**Methods:**

The systematic review reporting followed the Preferred Reporting Items for Systematic Reviews and Meta-Analysis (PRISMA) checklist. The literature was searched in PubMed, CINAHL, PsycINFO, Science Direct and Scopus databases. The identified records were screened using Covidence software, and data were extracted from included records using a predefined template. The mixed methods appraisal tool was used to assess the quality of the included studies. The spectrum of stakeholder engagement provided by the International Association for Public Participation (IAP2) was used as a guiding framework for synthesising the evidence related to stakeholder engagement.

**Results:**

A total of 1473 records were identified through the initial search after removing the duplicates. Twenty-six studies were included in the final review. The review results related to service users and other stakeholders’ engagement are presented under three overarching themes: (i) Methods and contexts of stakeholders’ engagement, (ii) Outcomes of stakeholders’ engagement, and (iii) Facilitators and barriers to stakeholders’ engagement.

**Conclusion:**

Various participatory approaches were utilised to engage the service users and other stakeholders in improving MNH service uptake and delivery. A wide range of service user- and provider-led outcomes were identified due to stakeholder engagement.

**Trial registration:**

**PROSPERO registration number:** CRD42022314613.

## Introduction

Globally, the engagement of service users and other stakeholders has been practiced as a joint decision-making process in improving health policy and services [1,2]. There is a common understanding among researchers, practitioners, and policymakers that stakeholder engagement (SE) in health services research is crucial to ensure the relevancy and usability of the jointly produced evidence [3,4]. Maternal and newborn health (MNH) service users in this review typically refers to pregnant women and postnatal mothers, whereas other stakeholders broadly include a wide range of entities, including but not limited to service users, family members (including partners of service users), health providers, policymakers, researchers, community members, civil society, leaders and health-related government and non-government organisations [5]. The World Health Organization (WHO) have also explicitly emphasised the importance of stakeholder engagement in maternal and newborn health (MNH) for empowering women, developing inter-sectorial collaborations, initiating joint learning, implementing people-centred MNH services and revitalising the ownership of MNH initiatives [6]. However, meaningful engagement of stakeholders in MNH improvement is often lacking in resource-poor settings [6]. Meaningful engagement of stakeholders often refers to the engagement of stakeholders in a range of processes and activities within an enabling environment where power is transferred to those who are affected by the decisions and actions related to MNH service [6,7]. This process helps to capture the diverse perspectives of stakeholders, empowering them to contribute actively and create awareness about their responsibilities in enhancing the healthcare policy and services. The stakeholder engagement process is broadly embedded within the principles of participative democracy, inclusivity, social justice, public accountability, and transparency [8]. Effective stakeholder engagements foster learning and innovation, identifying new approaches and ultimately contributing to sustainable health solutions [6,8].

According to the International Association for Public Participation (IAP2), stakeholder engagements can happen at five different levels: inform, consult, involve, collaborate, and empower [9]. Stakeholder engagements have been practiced at different levels in health needs identification, prioritisation, development, implementation, and evaluation of solutions [7]. Depending on the context, different terminologies have been used to refer to stakeholder engagement. For this review, we considered any terminology related to stakeholder engagement that meets the criteria of stakeholder engagement described by IAP2 [9]. We have not included ‘inform’ (the first level of stakeholder engagement) outlined in the IAP2 spectrum of public engagement as it does not fit with our definition of stakeholder engagement, which is basically a process of creating a shared understanding and being involved (two way) in decision-making [7]. Therefore, we included studies that depicted service users’ and other stakeholders’ engagement activities and aligned with the criteria of consult, involve, collaborate, and empower [9].

### Why is this review important?

The current progress in MNH in low and middle-income countries (LMICs) has been concerning in terms of meeting the Sustainable Development Goals (SDGs) targets 3.1 and 3.2. [6,10]. Multiple circumstances, such as a complex socio-cultural environment, shortage of skilled human resources, poor access to quality MNH services, fragile healthcare system, and lack of adequate quality evidence to make contextual decisions, have posed challenges in improving MNH services in LMICs [10,11]. With an aspiration to improve MNH services, there have been several attempts of different innovative efforts in engaging service users and other stakeholders in defining the problems and developing and implementing pragmatic solutions in different contexts within LMICs [12–15]. Nonetheless, in many cases, the practice of stakeholder engagement has often been symbolic and focused on meeting the funder’s requirements [16]. In this context, stakeholder engagements have been found to be frequently directed by professionals, with limited autonomy permitted to service users and other stakeholders. Such criticism and dispersion of the evidence have urged the need for compiled and synthesised evidence to elucidate the comprehensive landscape of stakeholder engagements in MNH services research in the context of LMICs. This synthesised evidence is expected to fill the gaps of evidence that potentially guide the way forward to the effective engagements of multiple stakeholders in MNH and, thereby, contribute to the efforts to achieve maternal and child health health-related targets in SDGs [6]. Therefore, this systematic review aims to review and synthesise the different practices for stakeholder engagements, characteristics of stakeholder engagements (e.g. contexts, diversity of stakeholders and levels of stakeholder engagement) and outcomes of stakeholder engagements in improving the MNH services uptake and delivery in the context of LMICs.

## Methods

The reporting of this systematic review was guided by the Preferred Reporting Items for Systematic Reviews and Meta-Analysis (PRISMA) checklist [17]. The adherence to the PRISMA checklist was aimed at incorporating essential components aligned with the systematic review methodology (S1 File). The systematic review protocol for this review was registered in PROSPERO (registration number CRD42022314613), and the detailed protocol has been published [18].

### Search strategy and information sources

The literature search was conducted in PubMed, PsycINFO, Scopus, Science Direct, and CINAHL databases using a search strategy that combined relevant keywords, controlled vocabulary, and Medical Subject Heading (MeSH) terms (S2 File). These search concepts were further expanded to include other sub-concepts/ MeSH terms. Combinations of search terms were modified for different databases. All identified indexed results were exported to Endnote software, and the duplications of the papers were checked and removed. Further, the final Endnote files of identified records from each separate database were transferred to Covidence software for screening. The hand search of relevant literature was also performed through a bibliography search of relevant retrieved papers. The full PubMed search syntax is provided in the supplementary file as an example of the search process (S2 File).

### Criteria for considering studies for this review

This review included peer-reviewed articles written in English and published between January 1990 and December 2023, since the United Nations supported the global maternal health initiative, and the global consensus on the need for wider stakeholders’ efforts to reduce maternal and child health mortality was jointly agreed upon in 1990, laying the foundation for the recognition of maternal and child health agenda in the Millennium Development Goals [19]. Similarly, the papers that have evidence related to service users’ or stakeholders’ engagement activities aimed at improving MNH service delivery and uptake in the LMICs (as per the World Bank classification) context were included in the review [20]. However, articles mentioning the conducting of formal training and continuing professional development (CPD) courses for health providers as stakeholder engagement activities for MNH improvements were not considered and have been excluded from the review. Qualitative, quantitative, and mixed-method studies were included in this review.

### Screening, study selection and data extraction

**Fig 1:**
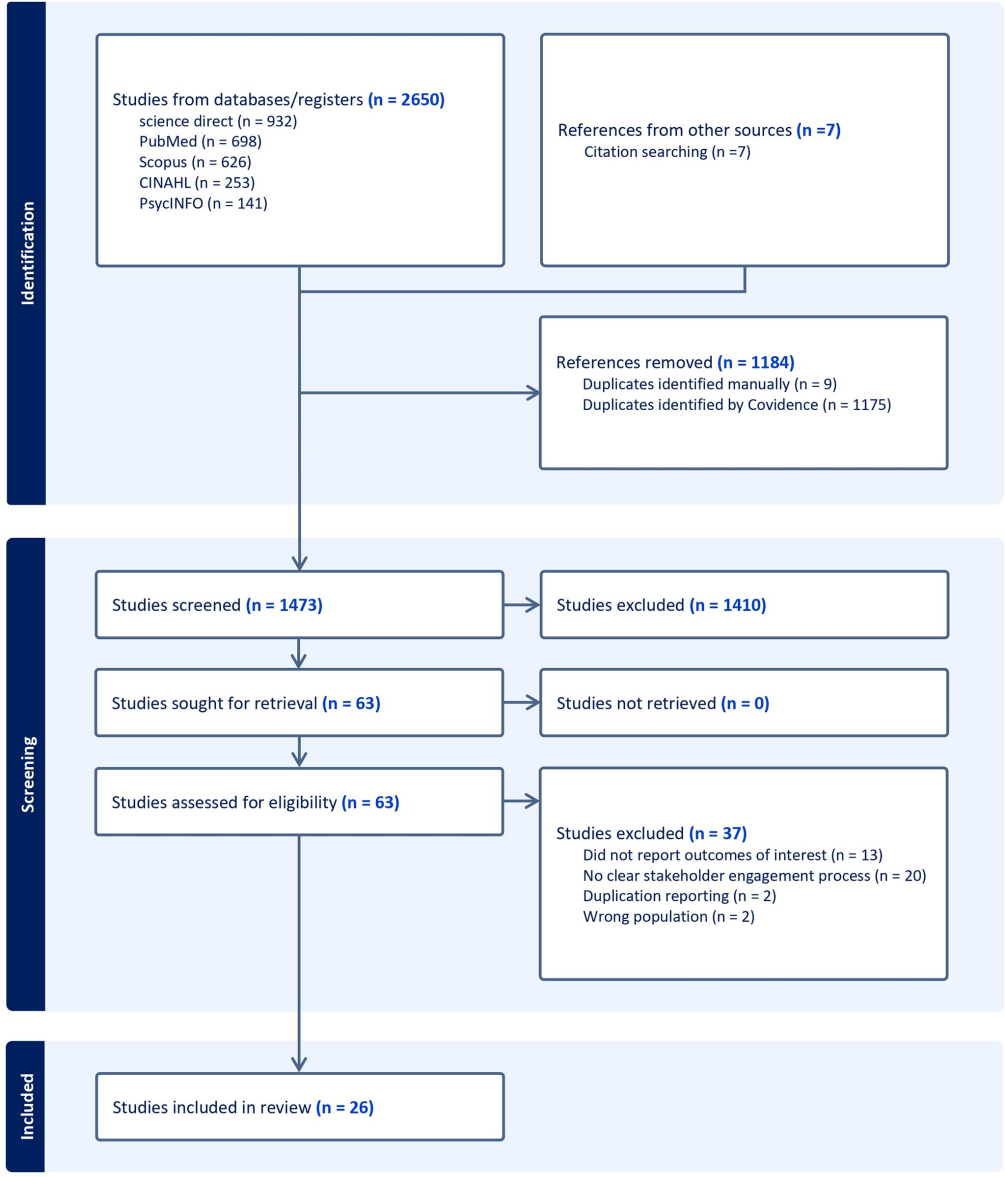
Preferred Reporting Items for Systematic Reviews and Meta-Analyses (PRISMA) flow diagram.

Using the Covidence software, two reviewers (DRS and RKS) independently screened the titles and abstracts of the identified articles. Further, the full-text review of the potential papers was also performed by two authors (DRS and RKS) to determine their eligibility for inclusion in the review (S3 File). Any discrepancies were resolved by discussion between two reviewers and, when necessary, consulted with other authors during the title and abstract screening and full-text review process. The first author (DRS) extracted the data into the structured template. The data was extracted under the headings of author(s)/data, study design, study settings, type of stakeholders, stage of stakeholder engagement, purpose of stakeholder engagement, process of stakeholder engagement and outcomes of stakeholder engagement (S4 File). The cross-verification of the extracted data was done by the second author (RKS). The two most common reasons for excluding the papers were wrong outcomes reported, the stakeholder engagement process not being defined clearly, and the wrong study population and duplication of study reporting. A PRISMA flow diagram shows the study selection process (Fig 1).

### Quality assessment

The quality assessment of the included papers was conducted by two reviewers (DRS and RKS) using the Mixed Methods Appraisal Tool (MMAT) [21]. The MMAT is a critical appraisal tool that is designed for the appraisal of qualitative, quantitative and mixed-method studies. The specific sets of quality assessment criteria (five characteristics for each group of study design) in the tool were followed as instructed in the tool. The quality of the papers was categorised as ‘low’, ‘medium’, and ‘high’ based on the overall quality of the papers. Those studies meeting four to five quality assessment criteria in the study were considered “high quality”, meeting three criteria was deemed as “medium quality”, while those studies meeting one or two assessment criteria were considered “low quality”. Any disagreement in the quality assessment marking was resolved by discussing it with the third author. Considering the quality assessment criteria of the MMAT tool, out of the total included studies, nine studies were considered high-quality, thirteen were considered medium-quality, and four were categorised as low-quality. The results of the quality assessment of the included papers are provided in (S5 File).

### Data synthesis

A narrative synthesis was used to combine the results from the studies included in this review [22]. Data synthesis was performed in two steps as guided by the Cochrane Handbook of Systematic Reviews [23]. At first, qualitative and quantitative evidence from all the selected articles was reviewed separately. Consequently, all types of results were combined into overarching themes through narrative synthesis. The integrated synthesis of the results is presented under the relevant themes identified during the results synthesis process. The main findings from each study are presented in standardised tables facilitating a systematic summary through tabulation, data transformation, common practice grouping, and textual descriptions.

## Results

A total of 1473 records were identified through the initial search after removing the duplicates. After the title and abstract screening of these records, 1410 records were excluded for not meeting the eligibility criteria. The remaining 63 papers were retrieved and included in the full-text review. Ultimately, 26 records that met the inclusion criteria were included in the final review.

### Characteristics of the included studies

The studies included in this review were conducted in 13 countries (Bangladesh, Burkina Faso, Democratic Republic of the Congo, Ethiopia, Ghana, India, Kenya, Mozambique, Nepal, Pakistan, Tanzania, Uganda, and Vietnam), encompassing both rural and urban contexts, including community and health facility settings (Table 1). Among the total 26 studies included in the review, 17 studies used experimental designs [12,24–38], five studies used qualitative methods [39–42], and four studies used mixed method design [15,43–46]. The most common types of stakeholders engaged in the studies reported were service users (pregnant women and postnatal mothers), male partners/husbands of service users (also referred to as fathers), community volunteers, female community health volunteers, women groups, and married women of reproductive age (which may include service users), household decision makers, mothers-in-law, health workers (nurses, midwives, doctors and community health workers), community health promoters/educators, local leaders, religious/faith-based leaders, artist/musicians, health activists, youth groups, local non-government organisational partners, and other government and non-nongovernment authorities at a different level of health structures. In terms of diversity in the engagements of stakeholders, 16 studies were found to have engaged service users (pregnant women and postnatal mothers) along with other stakeholders[12,14,15,24–26,28,29,32,33,35,40,41,43–46] and 10 studies engaged stakeholders other than service users to improve maternal and newborn health services [27,30,31,33,34,36–39]. In seven studies, male partners/husbands/fathers were considered key stakeholders for MNH improvement, and they were engaged in different ways in improving maternal and newborn health [14,31,35,40,41,44,46].

**Table 1:** Characteristics of the included studies.

Author (Date)	Study design	Country/ Context	Level of SE	Stage of SE	Stakeholders	Purpose of SE	Outcomes of SE
Alhassan et al. (2019) [34]	Experimental	Ghana (Community and health facility setting)	Empower	Development, implementation, and evaluation	Religious/faith-based groups; trader’s groups; widows’ group; community volunteers’ groups; musician groups; artisans’ groups and youth groups.	Community engagement intervention to improve MCH services utilization.	• Improved the utilisation of maternal and child health services.
Amosse et al. (2023) [31]	Experimental	Mozambique (Community setting)	Collaborate	Implementation	Male partners, in-laws, and community-level decision-makers, activist, local leaders, community health workers, and local health workers including nurses.	Community engagement to improve care-seeking behaviours and reduce maternal, perinatal and neonatal mortality and morbidity through a community-based intervention building capacity of community health workers	• Maximised the coverage of MNH messages among the target groups.
Azad et al. (2010) [30]	Experimental	Bangladesh (Community setting)	Empower	Development, implementation, and evaluation	Women group and local female facilitators and health workers	Engagement of local female facilitators to actively engage and empower women groups, assisting them in identifying maternal and neonatal issues and its solutions	• Facilitators’ use of participatory techniques stimulated community mobilisation in improving MNH.
Baker et al. (2018) [39]	Qualitative	Tanzania (Health facility)	Collaborate	Implementation	Health workers and other external organizational partners were	Stakeholders’ engagement to increase coverage and quality of essential evidence-based interventions for MNH.	• Collaborative quality improvement components were well understood and supportive in everyday practice, encompassing a strong alignment of health topics with local priorities, the use of run-charts for progress monitoring, and mentoring and coaching in individual health facilities.
Bich et al. (2014) [35]	Experimental	Vietnam (Community health centre, community, and household)	Involve	Development and implementation	Fathers, health workers and health educators, campaigners, and mothers	To motivate fathers to engage and take action in enhancing knowledge and motivation of mothers towards breastfeeding and newborn care.	• Fathers’ involvement has been found to influence early breastfeeding practices and reduce prelacteal feeding practices
Datiko et al. (2019) [15]	Mixed method	Ethiopia (Community and health facility setting)	Consult	Development and implementation	Health extension workers (HEWs), researchers, local female residents, HDA and PWFs	Enhance community engagement to improve MNH service utilisation and quality improvement.	• Increased engagement of HDA leaders, participation of pregnant women in PWFs and contributed to increased utilization of maternal health services.
Dhital et al. (2019) [24]	Experimental	Nepal (Community and health facility setting)	Collaborate	Implementation	Mothers of children below 12 months of age, local health promoters (a nurse, health assistant or auxiliary nursing midwife) and FCHVs	To rebuild the community health system through a participatory approach involving the local stakeholders and resources to improve the access to health care for mothers affected by the earthquake.	• Positively encouraged disaster-affected mothers, enhancing service utility, their knowledge and behaviors related to MNH
Ekirapa-Kiracho et al. (2016) [40]	Qualitative	Uganda (community setting)	Collaborate	Development, implementation, and evaluation	Local leaders, Village Health Team, Local transporters, Savings groups, pregnant women and their spouses, community development officer, Nurse or Clinical Officer, Religious leader, program implementers, facility managers, district health officers.	Aims to improve maternal and newborn health by increasing community awareness, action, and access to MNH services through multi-stakeholders’ engagement.	• Increased awareness about birth preparedness • Improved newborn care practices • Enhanced male involvement in MNH • Enhance collaboration to provide transport facilities for pregnant women. • Conducted home visits twice during pregnancy and twice in the first week after delivery • Demonstrated feasibility of supporting existing stakeholders and utilising community resources for MNH improvement
Fottrell et al. (2013) [33]	Experimental	Bangladesh (Community setting)	Empower	Development, implementation, and evaluation	Local women groups	Participatory women’s group intervention with higher population coverage on neonatal mortality reduction in Bangladesh.	• highly cost-effective approach to improve newborn survival and improve health behavior indicators in rural Bangladesh
George et al. (2018) [45]	Qualitative	India (Community and health facility setting)	Consult	Implementation	pregnant and postpartum women, community members, health providers, health authorities, NGO partner	Capacity building to improve care seeking and service delivery of maternity services for marginalised communities.	• Enhance understanding, trust, and collaboration across various health system levels • Increased awareness of MNH and improved utilisation of antenatal, institutional deliveries among marginalised populations • Enhanced accountability of services
Goudar et al. (2015) [25]	Experimental	India (Household-Community and hospital)	Empower	Development and implementation	Pregnant women, decision maker at household, health professionals	To test a composite intervention package designed to engage community members and health authorities to improve quality of care.	• Improvement in neonatal survival • Increase in transportation and facility delivery • Increase in the rate of facility births
Hoodbhoy et al. (2021) [14]	Experimental	Pakistan (Community setting)	Consult	Development and implementation	Male stakeholder, pregnant women and their families, female community health workers known as Lady Health Workers, local political leaders, and religious leaders.	Change in birth preparedness and complication readiness and pregnant women’s knowledge about pre-eclampsia as part of community engagement activities.	• Improve knowledge regarding complications of pre-eclampsia and health seeking behaviour in low-resource settings
Mafuta et al. (2017) [41]	Qualitative	Democratic Republic of the Congo (Community setting)	Involve	Development and implementation	Men, women, community health workers, representatives of the health sector and local authorities.	To develop a social accountability intervention that aims to improve maternal health services responsiveness and performance.	• Utilisation of the Dialogue Model for participatory engagement of beneficiaries • Articulation of suggestions on social accountability intervention components addressing core elements: voice, accountability and responsiveness
Kavi et al. (2022) [43]	Mixed method	India (Community and health facility settings)	Involve	Development and implementation	Pregnant women and women of reproductive age, mothers and mothers-in-law, community stakeholders and male household decision-makers and health workers	Community engagement in improving pre-eclampsia knowledge, birth preparedness and complication readiness, pregnancy-related care seeking and maternal morbidity.	• Household decision-makers gained awareness for pregnant women and enhancing the value of attending antenatal care. • Higher levels of birth preparedness observed • Positive shift in decision-makers views, making it easier for women to seek antenatal care without permission challenges
Maluka et al. (2023) [44]	Mixed method	Tanzania (Community setting)	Empower	Development, implementation, and evaluation	Women Groups (WGs), Male Champions (MCs), Women Group Supervisors (WGS), village leaders, community gatekeepers and health care providers	Participatory women groups engagements to improve MCH in resource-constrained settings.	• Increased the uptake of MCH services, enhance community empowerment and knowledge, and improve male involvement in MCH
Manandhar et al. (2004) [26]	Experimental	Nepal (community setting)	Collaborate	Development and implementation	Local Female facilitator, female community health volunteer and mothers’ groups (Married women of reproductive age)	Engage female facilitator supported women’s groups through an action-learning cycle to identify local perinatal problems and formulate strategies to address them.	• Reduced perinatal mortality, maternal mortality and improved antenatal care, institutional delivery
Mwaniki et al. (2014) [27]	Experimental	Kenya (Health facility setting)	Empower	Development and implementation	Local health workers and managers	Collaborate to empower local health workers and managers to improve quality of maternal and child health services	• Reduction on client waiting time • Improve supply of iron and folic acid tablets • Initiated regular structured dialogues with various community groups • Privacy during delivery was enhanced.
Morrison et al. (2020) [12]	Experimental	Nepal (Health facility and community settings)	Empower	Development, implementation, and evaluation	Female community health volunteers, women groups, local leaders and health workers.	Local stakeholder engagement to strengthen health management committees and enhance public accountability for MCH services.	• success in supporting communities to reduce institutional barriers to maternal care seeking
Pallangyo et al. (2018) [42]	Qualitative	Tanzania (Health facility)	Consult	Development and implementation	Nurse midwives, health care providers and leaders	Initiate facilitation strategy to improve postpartum care in a low-resource suburb	• Enhance implementing evidence and improving quality of postpartum care • Context-specific actions taken by the facilitators and healthcare providers
Thapa et al. (2019) [28]	Experimental	Nepal (Health facility and community settings)	Involve	Development and implementation	Nurse midwives, pregnant women	an adaptation of a group antenatal care model delivered by community health workers and midwives	• Enhance the experience of women antenatal care visits • Improve in knowledge of key pregnancy danger signs
Tripathy et al. (2016) [29]	Experimental	India (Community setting)	Empower	Development, implementation, and evaluation	Postnatal mother and health activist	Community-based strategies to improve maternal and newborn health through ASHAs.	• Reduce neonatal mortality and improve MNH service uptake
Persson et al. (2013) [32]	Experimental	Vietnam (Community and health facility)	Empower	Development, implementation, and evaluation	MNHG, physician, midwife, nurse, village health workers, local leaders, women union group.	Local stakeholder groups engagement in problem-solving approach	• Increased attendance to antenatal care and reduced neonatal mortality.
Hounton et al. (2009) [36]	Experimental	Burkina Faso (Community and health facility)	Consult	Development and implementation	Traditional leaders, Local associations Religious and political leaders	Engagement of local traditional leaders, administrative and religious leaders, local associations with the use of skilled care through problem-solving techniques, negotiation, and persuasion and lobbying to improve MNH access and uptake.	• Increased knowledge, preparedness for delivery, utilization of maternal health services. • Reduction in maternal, neonatal, and perinatal mortality
Tancred et al. (2018) [46]	Mixed method	Tanzania and Uganda (Community and health facility)	Empower	Development and implementation	Community members and local health service providers	Community members engagement in quality improvement of MNH services	• Change social norms around MH at the village levels
Hossain and Ross (2006) [38]	Experimental	Bangladesh (Community and health facility)	Consult	Development	Elected Union Parishad women members, leaders, active TBAs or village doctors, religious leaders, leaders from other sectors	Design to improve the quality of care	• Increased utilisation of EmOC services through community mobilization activities. • quality of care improved
Ahluwalia et al. (2003) [37]	Experimental	Tanzania (Community and health facility)	Involve	Development and implement	community leaders and village health workers	Village leaders was engaged in conducting community-wide meetings to discuss, refine, and implement the transportation and VHW support plans for their communities.	• More women with obstetrical problems used the community-based transport system • Retention of Village health workers

SE: Stakeholder engagement.

In the process of improving the access and utilisation of MNH services, in four studies, service users and other stakeholders were engaged specifically during the implementation stage of MNH programs [24,31,39,45]. Fourteen studies engaged service users and other stakeholders in the development and implementation stages of MNH programs/interventions [14,15,25–28,35–38,41–43,46]. Likewise, nine studies involved service users and other stakeholders across the development, implementation, and evaluation stages, focusing on introducing new MNH service initiatives or improving access and uptake of the existing MNH services [12,29,30,32–34,40,44].

### Themes

The key findings of the review are presented under three broader themes: (i) Methods and contexts of stakeholders’ engagement, (ii) Outcomes of stakeholders’ engagement, and (iii) Facilitators and barriers to stakeholders’ engagement.

#### i) Methods and contexts of stakeholders’ engagement

**Fig 2:**
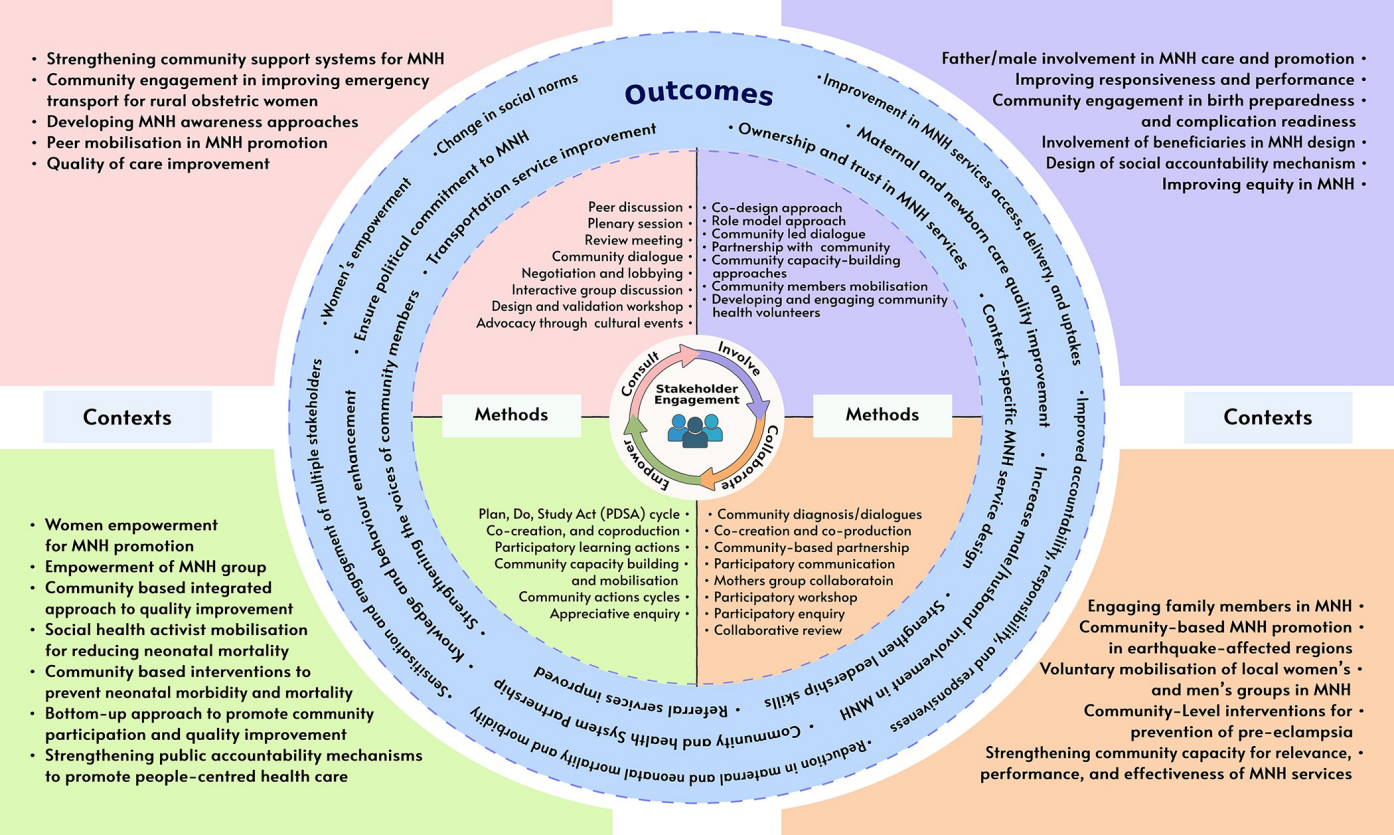
Methods, contexts, and outcomes of stakeholders’ engagement in maternal and newborn health services research.

This theme highlights the different approaches and levels of stakeholder engagement in improving MNH services uptake and delivery. According to the IAP2 Public Participation Spectrum, the level of stakeholders’ engagement in six studies was found to practice ‘Consult’ as stakeholder engagement [14,15,36,38,42,45], five studies practiced ‘Involve’ as stakeholder engagement [28,35,37,41,43], five studies practiced ‘Collaborate’ as stakeholder engagement [24,26,31,39,40], and ten studies practiced ‘Empower’ as stakeholder engagement [12,25,27,29,30,32–34,39,46] in the process of improving the delivery and uptake of maternal and newborn health services (Fig 2).

*‘Consult’ as engagement*. The ‘Consult’ level of engagement actions as stakeholders’ engagement processes were employed through various approaches in the studies to collect service users’ and other stakeholders’ opinions, feedback, and suggestions in the development, implementation, and evaluation of MNH services. In several studies included in the review, some common techniques used for consultation among multiple stakeholders were conducted using interactive group discussions, consultative meetings, and workshops [14,15,25,38,42,45], community monitoring and dialogue with health authorities [38,42,45]. The consultation also involved interactive sessions on MNH issues with male stakeholders such as husbands, social workers, political activists, religious leaders, and landlords of local communities [14,42]. In the study conducted in Burkina Faso, local traditional leaders, community relay workers, and administrative and religious leaders were consulted and engaged through workshops, group discussions, plenary sessions, application of problem-solving techniques, negotiation, and persuasion and lobbying as a community engagement strategy to improve MNH services [36]. There was also the animation of traditional music concerts for advocacy for safe delivery through wider community influencers’ engagements [36]. The community consultation strategy in this study was inspired by the sense that communities are not ‘empty vessels’ and understanding and building upon local cultural beliefs of MNH care is crucial for effective communication of MNH issues [36]. In Bangladesh, a study reported that stakeholder committees were formed to solicit community opinions and suggestions for improving MNH service provision [38]. The stakeholder committee comprised community members, elected Union Parishad women members, leaders, trained traditional birth attendants (TBA) or village doctors, religious leaders, and leaders from other sectors such as educational institutions and community-based organisations. The stakeholder committee regularly monitored health facility cleanliness, collected client feedback on services, and participated in reviewing maternal death or near-miss cases [38].

*‘Involve’ as engagement*. The ‘Involve’ as engagement strategies reported in the studies were found to be performed through the involvement of different leaders, beneficiaries, local community health workers, and health committees as intermediaries for gathering community concerns related to MNH services and involved through different actions for enhancing MNH services [28,35,37,41,43]. The involvement actions included capacity building of service users, their male partners/husbands, and community members to raise their voices and support MNH activities [28,35,37,41,43], developing community partnerships around social accountability in MNH through community dialogue [37], and sensitisation workshops among health providers to enhance the capacity to understand users’ voices and promote the practice of social accountability [41]. For example, in rural Tanzania, community members participated in the capacity-building meeting to discuss ways of mobilising the community to establish local transportation services for mothers with obstetric emergencies [37]. As part of community capacity building and engagement, village health workers were locally recruited and mobilised to improve MNH outcomes in rural areas by bridging the gap between communities and formal healthcare systems [37]. Pregnant women in Nepal were involved in the MNH promotion activities through service users-led facilitated peer discussions during ANC visits [28]. Of note, one study from Vietnam engaged male partners/husbands/fathers to promote MNH through role model approaches, where the concept of the "Fathers’ Contest” was designed as a community mobilisation strategy to motivate fathers’ involvement in exclusive breastfeeding and support in MNH service uptake [35]. Farmers and the community organised the contest in the name of "Who loves their wives and children more?". Those fathers demonstrating praiseworthy care for their wives and infants were selected for awards and titles.

*‘Collaborate’ as engagement*. Likewise, the level of engagement denoted as ’Collaborate’ in the papers included in the review embraced the participatory approaches to identify problems, develop solutions, and implement the solutions through the adaptation of different techniques such as participatory workshops, participatory enquiry, community diagnosis/dialogues, co-creation approach, collaborative review meeting, participatory communication (talk shows and radio programs), and collaborations with local volunteers and community-based partners in the implementation of interventions [24,26,31,39,40]. For instance, two studies from Nepal highlighted collaborative efforts as a process for enabling women for MNH through a community health engagement process [24,26]. This involved the engagement of local women as health promoters or facilitators and female community health volunteers to implement the change. These local females played a crucial role in enhancing access and uptake of MNH services by facilitating participatory mother group meetings in the community [24,26].

In Mozambique, the co-creation approach was utilised as a community engagement strategy to involve and collaborate with stakeholders such as male partners, mothers-in-law, community-level decision-makers and other stakeholders for developing education materials, improving transportation services for mothers, and implementing co-created products to enhance health care-seeking behaviours and improving access and uptake of MNH services [31]. In a study from India, service users and their family members collaborated with local service providers to enhance learning, prepare effective birth plans, and practice home-based life-saving skills [25]. In studies from rural Uganda, the participatory inquiry technique was employed to enhance community stakeholder capabilities through mobilisation and support mechanisms[40].

*‘Empower’ as engagement*. Service users, local health service providers, and community members were empowered and mobilised to enhance MNH service delivery and utilisation. ‘Empower’ level of engagement techniques commonly practised to empower the service users and other stakeholders included community-based participatory approaches such as participatory learning and action cycle, Plan, Do, Study, Act (PDSA) cycle and run charts, Community Action Cycle (CAC), Four ‘D’ Cycle of Appreciative Inquiry (Discovery, Dream, Design, Deliver), co-production, and co-creation approaches [12,25,27,29,30,32–34,39,46]. These participatory approaches identified under this sub-theme were utilised to empower pregnant women and postnatal mothers, women’s groups, local female health promoters/volunteers, health workers, leaders, and other community members by bringing their voices and collaborative efforts in planning and cyclically evaluating MNH actions. For example, in the studies from Tanzania and Kenya, stakeholders strengthened their capacity to facilitate a structured process of problem-solving and testing of change ideas for prioritised quality improvement issues, with the progress systematically monitored by themselves over time using run charts techniques [27,39]. Similar approaches were also used in Uganda and Tanzania, where community volunteers were trained and were one of the key actors within the local health system to improve community-level MNH efforts [46]. Health volunteers were taught to identify MNH issues in their communities and then encouraged to continuously assess the feasibility of improvement of MNH services [46]. Similarly, in a study conducted in Vietnam, local female health promoters used the PDSA cycle to mobilise and empower Maternal and Newborn Health Groups (MNHG) in identifying and prioritising local perinatal health problems, implementing improvement cycles and continuously addressing various issues related to MNH in volunteer groups [32]. The MNHG included community healthcare staff, village health workers, community collaborators, and women’s union representatives.

This stakeholder’s engagement process enhanced their knowledge, skills, and confidence in decision-making by themselves. Similarly, a study from India showed community mobilisation efforts based on the Community Action Cycle (CAC) approach, which were employed to empower communities to actively identify, prioritise, and address MNH problems through collaboration with various community members, including service users, mothers-in-law, male partners/husbands, and community facilitators [25]. Studies from Bangladesh and India showed that local women’s groups were empowered to prioritise MNH issues and collaboratively design and implement strategies to address these identified concerns through participatory learning and action cycles [29,30,33]. Similarly, a study from Nepal highlighted the local stakeholder engagement to strengthen the capacity of health facility management committees, local women’s groups, and Female Community Health Volunteers (FCHVs) to lead discussions on obstacles to institutional delivery and its possible solutions [12]. Likewise, a bottom-up approach to community engagement in Ghana empowered community members to assess MNH service quality on non-technical aspects like staff attitude, punctuality, feedback, information provision, and medication dispensation [34]. Further, the community MNH quality care champions then ensured the implementation of action plans in the health facilities [34].

#### ii) Outcomes of stakeholders’ engagement

This theme presents outcomes of stakeholder engagement for improving MNH services in different contexts. The different outcomes resulting from stakeholder engagement were found to be either led by service users or service providers. Those outcomes led by service users were found to be driven by their expectations, needs and preferences for improvements in MNH. This outcome was mostly observed regarding improvements in service users’ experiences, knowledge and attitudes related to MNH. Conversely, the outcomes led by service providers were mostly aligned with the improvements in MNH service delivery and attainments of institutional objectives. Therefore, the wide range of outcomes from stakeholder engagement identified in the included papers were briefly presented under two sub-headings: i) service user-led outcomes and ii) service provider-led outcomes.

i) *Service user-led outcomes*. Several studies in the review reported the wider engagement of service users and other community stakeholders empowered service users and community members and enhanced their MNH knowledge and behaviour [12,14,15,24,25,28,32–35,39,40,42–44]. Two studies from India showed that community engagement meetings involving pregnant women, household decision-makers, community stakeholders, and health workers improved women’s approaches towards receiving support from their families, facilitating easier access to antenatal care, arrangements for transport, permission for emergency care, and identification of a health facility for delivery [29,43]. Studies from Nepal highlighted the FCHV-led participatory intervention with rural women’s groups’ engagements improved antenatal care check-ups, institutional delivery, trained birth attendance, and hygienic care [12,24,26,28]. As part of participatory engagement, the community developed some innovative strategies to support mothers, such as community-generated funds for maternal or infant care, stretcher schemes, production and distribution of clean delivery kits, home visits by group members to newly pregnant mothers, and awareness raising with a locally made film to create a forum for discussion [26]. The increased engagement of Health Development Army (HDA) leaders in southern Ethiopia also led to improved utilisation of MNH services [15]. Study findings from Ghana suggested that bottom-up community engagement in MNH could be a novel strategy to enhance trust and confidence, particularly in a low-resource context [34]. Male involvement in the MNH was recognised as a "new strategy for old problems," where fathers’ involvement in supporting antenatal care had a positive impact on the overall uptake of MNH services and newborn care, including the early initiation of breastfeeding and reduced the use of pre-lacteal feeding [35,39].

Moreover, the participatory engagement of local women groups in MNH interventions in Bangladesh, India, Nepal, and Vietnam reported a considerable reduction in neonatal and maternal mortality [26,29,30,32,33]. The local women’s group mobilisation led to a considerable decrease in neonatal mortality by up to 38%, along with positive changes in home delivery practices, essential newborn care, and feeding practices [30,32,33]. Community-level volunteers such as the Accredited Social Health Activists (ASHA) programme in India and FCHVs in Nepal helped identify MNH-related health problems, prioritise the problems, and develop and implement strategies to address them at grassroots levels [26,29]. It was found to contribute to improving neonatal survival and reducing maternal mortality [26,29]. Likewise, community mobilisation facilitated timely access to emergency obstetric care and improved neonatal survival, transportation services, and facility-based deliveries [25]. In rural Tanzania, community-led initiatives helped establish a community transportation system for mothers with obstetric emergencies, which was developed due to local community stakeholder engagement efforts [37].

ii) *Service provider-led outcomes*. Several studies in the review reported that the engagement of multiple stakeholders at different levels and contexts was found to have a positive influence on improving the quality and delivery of MNH services. In Kenya, collaborative engagements of local MNH service providers supported and mentioned by the facilitation team helped to address performance gaps through rigorous examination, root cause identification, and the development and implementation of change ideas [27]. The initiative highlighted the successful improvement of healthcare quality in small rural facilities in low-income settings without additional external resources. It also highlighted the applicability of quality improvement principles to enhance access and adherence to standards at care points [27]. Similarly, a study from Uganda showed that the engagement of stakeholders using the community Dialogue Model involved community beneficiaries, particularly women; engaging with diverse stakeholders in a participatory advisory process enabled the participants to bring their voices to design the social accountability initiatives in improving MNH [41]. The initiative addresses the essential elements of voice, accountability and responsiveness in the context of community involvement [41]. Two studies from Nepal highlighted that strengthening health facility management committees and mobilising FCHVs successfully reduced institutional barriers to maternal care [12,24]. The engagement of local female health promoters enhanced the capacity of FCHVs to organise mothers’ group meetings, engage wider community stakeholders, and strengthen the local health systems [24]. Similarly, the study conducted in southern Mozambique reported that the MNH promotion had greater acceptance through service users and community engagement strategy [31]. The engagement of household decision-makers and community leaders helped to build local support for maternal health and adapt messages to local needs [31]. In Burkina Faso, various consultation techniques for engaging stakeholders were utilised to reach a consensus and achieve community support for improving and utilising MNH services [36].

Moreover, two studies from Tanzania showed the wider stakeholders’ engagement in the contributions to MNH services’ quality improvement [39,42]. Collaborative learning sessions, mentoring, and coaching enhanced health workers’ knowledge and skills in quality improvement topics, boosting motivation and capacity to implement quality improvement (QI) using PDSA cycles and run charts [39]. The regular community dialogues discovered concerns about healthcare staff attitudes, lack of privacy during delivery, denial of trusted companions’ access, insufficient facilities for post-delivery care, and a lack of food [27]. Likewise, in another study, the facilitator-based initiative postpartum care quality improvement initiative was achieved by employing diverse stakeholder engagement strategies that fostered an empowering and collaborative work approach [42]. The participatory approach strengthened providers’ leadership skills and enabled them to optimise utilising the limited local resources to produce quality MNH services [27].

#### iii) Facilitators and barriers to stakeholders’ engagement

Though not all the papers included in the review have documented barriers and facilitators from their formal evaluation of the stakeholder engagements process, a wide range of barriers and facilitators were recognised. The facilitators for the stakeholder engagement included decentralised health governance structures, which enhanced the decision-making process for the management of local stakeholders’ involvement of local leaders and their aspiration to demonstrate leadership and public accountability in the community to create a favourable environment for accelerating the stakeholder engagement process at the grassroots level [12,14,15,31,32,37,40]. Proactive engagement from the technical team in negotiating with local leaders showed a unique and impactful approach to quality improvement, jointly with wider stakeholders’ engagement [42]. In addition, the local non-governmental organisations (NGOs) with extensive experience in selected communities played effective roles in bringing stakeholders together, establishing collaboration and helping foster relationships of trust and credibility with local community members [24,26,45]. Mobilisation of formal and informal social structures enabled continuing stakeholder engagements in MNH [37]. Institutional leadership support was crucial in facilitating implementation efforts at institution-based initiatives [12,27,42]. Reaching service users through community resource persons and regular structured dialogues with communities enhanced both engagement and service utilisation [27].

In countries that have already established community volunteer systems, such as FCHVs in Nepal [12,24,28], Accredited Social Health Activists in India [43], Village Health Workers in Vietnam [32], Health Development Army in Ethiopia, Lady Health Workers in Pakistan [14], Community Health Workers in Democratic Republic of the Congo [41], Community relay workers in Burkina Faso [36], and similar types of volunteers in other countries contributed towards facilitating the stakeholder engagements in MNH improvement process in different ways. However, supervision was crucial to motivate community health volunteers and retain their participatory process in MNH initiatives. The demand-driven comprehensive community partnership approach with a better understanding of the social structure of the local context, the political and cultural logic of power and the ownership of the stakeholder engagement process were instrumental for the meaningful engagement of diverse stakeholders in MNH improvement [36,38]. The use of participatory learning action cycles by local volunteers enabled their responsiveness to work actively on context specific MNH issues [25,27,32,34,39,46]. Effective stakeholder engagements were also contingent on the participants’ identity and selection process of community representatives, which could serve as a platform for already-known groups or individuals to exert their influence.

On the contrary, the lack of a sense of personal/group responsibility and ownership towards initiatives among stakeholders, poor trust between the community and local health workers, unmet demand of financial accountability, ambiguity in the communities’ role, expectations, and obligations, inadequate understanding of policy processes and institutions (policy literacy), and inadequate local people’s commitment towards initiatives were noted as impeding factors for stakeholders’ engagement process [14,24,31,40]. Despite the priority for engagement of marginalised groups in certain studies, the effective engagement of such groups was hindered due to a lack of support from local political leaders, poor financial support and inadequate capacity-building systems [12,30]. The low representation from certain groups, particularly underrepresented or decision-maker groups, hinders the participants from openly engaging and sharing their views during meetings [31]. Male partners/husbands (i.e. fathers) exhibited less engagement in MNH activities, which was attributed not only to socio-cultural circumstances but also to constraining the availability of their appropriate time to participate, given their long hours of work shifts dedicated to familial support outside the home [31,35]. The stakeholders’ participation challenges were especially from rural and poor socio-economic backgrounds due to inadequate incentives, absenteeism, workload, and distance from the community for the stakeholder engagement activities [15,30]. Similarly, an unclear organisational structure, lack of clarity about team mobilisation, and inadequate resources to cover the workloads also hampered stakeholder engagement [42]. In some studies, gendered barriers included women feeling restricted in unions, joining groups, and actively engaging in service improvement actions despite their willingness [13,27,31,32].

## Discussion

This review contributes to the comprehensive documentation of stakeholder engagement in improving access and utilisation of MNH services in the context of LMICs, as a synthesis of such evidence has been lacking so far. Considering the complex nature of MNH issues and poor resource availability in LMIC contexts, diverse approaches have been attempted to engage service users and other stakeholders in identifying problems and developing, implementing, and evaluating solutions. This systematic review presents diverse applications of methodological approaches related to stakeholder engagements in MNH services in the context of LMICs. Notably, these stakeholder engagements were practiced both within the healthcare facilities and in the communities [47]. Also, the review provided evidence on outcomes of the stakeholder engagement and potential barriers and facilitators in the stakeholder engagement process in improving MNH. In this review, as per the IAP2 framework of stakeholder engagements [9], we identified different levels of stakeholder engagements of multiple stakeholders in MNH services, which happened at different levels within and outside health systems. Stakeholder engagement approaches in health services improvement initiatives are grounded on the belief that community engagement involvement ensures the development of context-specific MNH intervention originating from local knowledge and assures its accountability and sustainability [48–50]. For example, those studies which applied empowerment level of engagement explicitly showed alignment with the WHO definition of stakeholder engagements, which describes stakeholder engagements as a process of developing relationships that enable stakeholders to work together and gain access to processes for assessing, analysing, planning, leading, implementing, monitoring, and evaluating actions, programmes, and policies [6]. Other studies that followed consult, involve, or collaborate forms of stakeholder engagements did not clearly depict establishing relationships with stakeholders, continuing the process, or sustainability of the outcomes, as in most cases, the stakeholder engagements were a one-time event. Similar results were revealed in the review conducted to investigate community engagement in improving primary healthcare services for universal health coverage [48]. In most papers included in the current review, the stakeholder engagement process followed the pragmatic way of participatory approach in collecting community inputs for the development, implementation, and evaluation of MNH service initiatives/programs. However, the assurance of the diversity of service users and other stakeholders considering their disadvantaged and marginalised situation was less highlighted in the papers. It is widely emphasised that stakeholder engagements should facilitate the process of inclusiveness by ensuring the active participation of those who are not adequately represented or are left behind in society due to several social and structural barriers [3,49].

Also, a wide range of individual, community, and institutional outcomes were linked to the results of stakeholder engagements in improving MNH services’ quality, delivery, access, and uptake. However, the effectiveness of stakeholder engagements in MNH services requires understanding the approaches for meaningful engagements of stakeholders and practical procedures of stakeholder engagement, including stakeholder identification and engagement process [3,50,51]. The stakeholders in the included papers were broadly categorised into three groups, i.e. end users (service users), community members and service providers. However, there were limited descriptions of stakeholders’ mapping and selection procedures in the studies included in the review. The review reflects that in most resource-constrained settings, power dynamics predominantly exist within the providers or health care system, notwithstanding the praiseworthy space for the efforts made in the initiation of stakeholder engagements practice in MNH services in the LMIC context. The stakeholder engagement process, therefore, in those contexts was mostly bureaucratic or professional deliberation where service users and other stakeholders were consulted or involved but had limited practice of their autonomy. These results are consistent with the findings from the previous study, where symbolic consultation with local stakeholders was performed as part of a stakeholder engagement in an inclusive policy-making process [52]. This usually happens due to high socio-economic inequalities among stakeholders, stakeholder hierarchy, mutual distrust, no culture of stakeholder engagement, differences in communication skills, and self-confidence among the group members expected to work together as part of a stakeholder engagement process [49,52]. Thus, understanding the influence of contexts in the stakeholder engagement process is crucial as this shapes the participants’ identities and how they can exercise their power for interaction in a group [50,53]. For example, contextual factors such as decentralised health governance structures, public accountability of local leaders, and mobilisation of local networks and resources were found as some facilitating factors for stakeholder engagement in our review.

In most papers in the review, local women were found to actively participate as community volunteers, illustrating their supportive and empathetic stance towards their peers, particularly those in the perinatal period. Despite challenging circumstances marked by poverty and other adverse environmental conditions, women have demonstrated an unwavering commitment to assisting their female peers within the community [54,55]. The success of a significant proportion of these initiatives can be attributed to the dedicated involvement of female volunteers at the grassroots level, who exhibited consistent commitment devoid of any self-interest. In contrast, men were found to have limited engagement in enhancing MNH at the family and community levels. This phenomenon might be attributed to the sociocultural norms of certain societies where they perceive maternal and newborn healthcare as more associated with female roles and gendered spaces. Men often have to face different kinds of barriers even if they are willing to provide support in promoting maternal and newborn health, either due to poor knowledge about MNH or restrictions created due to socio-cultural factors [56,57]. Likewise, the local community-based NGOs were key organisational partners in playing the mediating role in facilitating stakeholder engagement in MNH. They demonstrated a substantial commitment to capacity building and facilitation, aiming to enhance mutual understanding, trust, and collaboration among diverse stakeholders operating across different health system levels in different contexts. The NGOs might have leading roles due to their local establishment, a better understanding of the community members, trust, and potential to build partnerships in joint initiatives and foster support for community actions [58].

Participatory learning and action tools, such as the PDSA cycle [59], CAC, and appreciative inquiry [60], were often used to empower women and other community members to identify the problems and implement and review the solutions. The enabling and inhibiting environment for effective use of such participatory tools in the process of stakeholder engagement could be influenced by the local community capacity in terms of their community leadership, governance, and management, and more specifically, with health knowledge, skills, and abilities [13,61,62]. Due to the low status of women, gender inequality, illiteracy among women, and other environmental circumstances, especially in the rural context, facilitators were recruited to regularly guide stakeholders in using tools to improve their active engagement. There might be challenges to adopting such stakeholder engagement techniques among stakeholders at the beginning. Still, a participatory way of engagement has been widely considered an effective technique for promoting stakeholders’ active and meaningful involvement [13,61]. Also, such participatory learning action approaches were shown to be effective for incorporating inputs from stakeholders at different stages of MNH initiatives. However, identifying specific outcomes resulting from stakeholder engagement in MNH is a complex task, which has also been acknowledged in previous studies [2]. Studies in Afghanistan reported a series of participatory approaches in improving overall health services access and utilisation at local health facilities [63,64]. The use of community scorecards and balanced scorecards was found to be a useful tool for community engagement in the generation of key performance indicators, voicing community and health provider concerns, improving transparency and accountability and joint decision-making to enable improvements in health services access and utilisation [63,64]. Thus, the outcomes of stakeholder engagement in health services improvement do not happen straightforwardly or linearly; rather, it is a complex process and is performed in a series of events and cycles [2,53]. Our review has reported the important evidence drawn from multiple papers on how different ways of stakeholder engagements in MNH have contributed to improving the service delivery and uptake in the context of LMICs. Given the different sociocultural, political, and other environmental contexts of stakeholder engagements, it was obvious that there was no such universal formula that ‘one size fits all’ could be applied in engaging stakeholders to improve MNH services. However, it was evident that context-specific meaningful stakeholder engagement activities were recognised as an essential approach to putting collective efforts together for realistic design, development, implementation, and review of the MNH initiative.

The effective stakeholders’ engagement in improving MNH in LMICs has significant policy implications. The evidence shows that the broader engagement of multiple stakeholders, including service users, family members, service providers, community members/networks, policymakers, and non-governmental organisations, could ensure the comprehensiveness of the MNH efforts at different levels. Likewise, MNH initiatives shaped by multi-stakeholders could have greater potential to address local needs, effective mobilisation of resources, and foster a sense of ownership and responsibility. Also, sustainable collaboration and coordination could happen through meaningful engagements of stakeholders at different stages of program and policy development and implementation.

### Strengths and limitations of the review

Since the outcomes of the stakeholder engagements in all studies were not based on a true control group and stakeholder engagement outcomes were not measured directly or linearly, it was not possible to interpret the strength or size of impacts that stakeholder engagement activities contributed to enhancing MNH. Given the heterogeneity of study designs, diverse contexts, stakeholders, and stakeholder engagement approaches, we could not pool the estimates of stakeholder engagement effects on MNH service improvement. However, as much as possible, we have presented the summarised forms of integrated and synthesised results related to methods, context, and outcomes of stakeholders’ engagement from the studies. This is the first review of its kind that has systematically reviewed the stakeholder engagement activities in MNH services in the LMIC context. The review has also managed to cover a wide range of databases. The insights drawn for the review are valuable evidence concerning different stakeholders in MNH, including service users and their families, policymakers, planners, and implementers in resource-constrained settings.

## Conclusions

Our review highlighted the different ways of stakeholder engagements in designing, implementing, and evaluating MNH initiatives and their contributions to improving MNH service delivery and uptake in LMICs. The service users and other stakeholders were engaged in MNH initiatives through various techniques at the Consult, Involve, Collaborate, and Empower levels of engagement activities. The future stakeholder engagement in MNH service research could benefit from using the IAP2 concepts of stakeholder engagements for better clarity in defining the scope, levels, and process for engaging stakeholders. Contextual factors such as settings, socio-political environment, diversity of stakeholders, socioeconomic background of service users and community members, and other environmental circumstances are crucial to understanding the effective engagements of stakeholders and producing optimum outcomes in MNH improvements. The participatory approaches of stakeholder engagement were found to facilitate the process that strives for transformative change for improvement in MNH. Applications of this process were found to emphasise the continuous engagements and learning for stakeholders, value diversity, support group interactions, and recognise the significance of context. Thus, using guidelines tailored to the local context considering socio-cultural dynamics and potentials of the health care system would enhance the transparency in the stakeholder engagement process, ensure the essence of stakeholder engagement requirements, and enhance the potential impacts and documentation of the important learnings. Stakeholder engagement in MNH needs to be adequately promoted in the context of LMICs, as it fosters collaboration, leverages local knowledge, and ensures that solutions are culturally relevant and sustainable, directly addressing the unique challenges faced by the communities.

## Supporting information

S1 File PRISMA checklist. (DOCX)

S2 File Search concepts and database search. (DOCX)

S3 File Studies identified for full text review. (XLSX)

S4 File Data extraction from primary source. (XLSX)

S5 File Risk of bias and quality assessment of studies. (DOCX)

## Abbreviations

ASHAs: Accredited Social Health Activists
CAC: Community Action Cycle
EmOC: Emergency obstetric care
FCHVs: Female Community Health Volunteers
IAPP: International Association for Public Participation
HDA: Health Development Army
INGO: International Non-governmental Organisation
LMICs: Low- and middle-income countries
MMAT: Mixed Methods Appraisal Tool
MNH: Maternal and newborn health
MNHG: Maternal and Newborn Health Groups
NGOs: Non-governmental Organisations
PDSA: Plan, Do, Study, Act
MCH: Maternal and Child Health
PWF: Pregnant women forums
SDG: Sustainable Development Goal
SE: Stakeholder engagement
TBA: Traditional Birth Attendant
VHW: Village Health Worker
WHO: World Health Organisation
