# Supplementary material for: Understanding service users and other stakeholders’ engagement in maternal and newborn health services research: A systematic review of evidence from low- and middle-income countries
Source: PLoS One. 2024 Nov 27;19(11):e0309888. doi: 10.1371/journal.pone.0309888 (PMC11602069; doi:10.1371/journal.pone.0309888)
Supplement: S2 File — (DOCX) [file pone.0309888.s002.docx]

**S2 File: Search concepts and database search**

**Literature search concepts and sub-concepts/MeSH terms**

| **Search concepts** | **Sub-concepts/ MeSH terms** |
| --- | --- |
| Service user | patient, client, consumer, mothers, husband, partner*, family, relative, “service user*”, “pregnant mothers”, “mother-in-law”, “caregiver”, “pregnant post postpartum women” “postnatal women” “lactating mother”, “new mother”, mother*, client*, ‘neonate’, and ‘newborn’. |
| Stakeholder | public, provider, manager, health worker, doctor, nurse, midwives, policymaker, policy implementer, health assistant, volunteer, advocacy group, organization, NGO, INGO, leader, community, “community leader*”, school, club, academia, researcher, committee. |
| Engagement | participation, participatory, involve*, “patient and public involvement”, PPI, collaborat*, engag*, partner*, “patient participation”, and user involvement”. |
| Types of research | community-based research, community-based participatory research, co-design, co-production, co-creation |
| Maternal and newborn health | “maternal health”, “newborn health”, “child health”, “antenatal service”, “antenatal checkup”, “institutional delivery”, “health facility delivery”, “postnatal checkup”, “postnatal service*”, and “newborn health”, “neonatal health”, “prenatal care”, “perinatal care”, and “maternal mental health”. |
| Middle and low-income countries | “developing country”, “low income countries”, “low and lower middle income countries”, “low and lower-middle income countries”, “underdeveloped countries”, third world, list of LMIC individual countries as per the World Bank published report of 2023 |

**Sample of database search**

**PubMed Query**

| **Search** # | **Query** | **Result** |
| --- | --- | --- |
| #1 AND #2 AND #3 AND #4 AND #5 AND #6 | (((((((((((((((((((patient) OR (mother*)) OR (consumer)) OR (client*)) OR (husband)) OR (pregnant mother*)) OR ("pregnant women")) OR ("mother in law")) OR (caregiver)) OR ("postpartum women")) OR ("lactating mother")) OR ("new mother")) OR (newborn)) OR (neonate)) OR (service user*) AND (english[Filter])) AND (((((((((((("maternal health") ) OR (newborn health)) OR (child health)) OR ("antenatal service")) OR (antenatal checkup*)) OR (institutional delivery)) OR (postnatal service*)) OR ("neonatal health")) OR ("prenatal care")) OR ("perinatal care")) OR ("maternal mental health") AND (english[Filter]))) AND ((((((((("community-based research") OR ("community-based participatory research")) OR (co-design)) OR (coproduction)) OR (cocreation)) OR (research)) OR (evaluation)) OR ("program plan*")) OR ("implementation research") AND (english[Filter]))) AND ((((((((("developing country") OR ("low-income countries")) OR ("low and lower-middle-income countries")) OR ("low and lower-middle income countries",)) OR ("low and lower-middle income countries")) OR ("underdeveloped countries")) OR (third world)) OR (LMIC)) OR (LMICs) AND (english[Filter]))) AND (((((((((((((((((((((((((stakeholder) OR (public)) OR (provider)) OR ("service providers")) OR ("health worker")) OR (doctors)) OR (nurse*)) OR (midwives)) OR (policymakers)) OR (policy implementer)) OR ("health assistant")) OR (volunteer)) OR ("advocacy group")) OR (organisation)) OR (organization)) OR (NGO)) OR (INGO)) OR (community leader*)) OR ("community member")) OR (school)) OR (club)) OR (academia)) OR (researcher)) OR (committee) AND (english[Filter])) AND (community- based research) AND (english[Filter]))) AND ((((((((((((engagement) OR (involvement)) OR (engage*)) OR (participation)) OR (participatory)) OR ("patient and public involvement")) OR (PPI)) OR (Collaborat*)) OR (partner*)) OR ("patient participation")) OR ("public involvement")) OR ("user involvement | **698** |
| #6 | (((((((((((engagement) OR (involvement)) OR (engage*)) OR (participation)) OR (participatory)) OR ("patient and public involvement")) OR (PPI)) OR (Collaborat*)) OR (partner*)) OR ("patient participation")) OR ("public involvement")) OR ("user involvement") OR (involv*) AND (english[Filter]) | 4568028 |
| #5 | ((((((((((((((((((((((((stakeholder) OR (public)) OR (provider)) OR ("service providers")) OR ("health worker")) OR (doctors)) OR (nurse*)) OR (midwives)) OR (policymakers)) OR (policy implementer)) OR ("health assistant")) OR (volunteer)) OR ("advocacy group")) OR (organisation)) OR (organization)) OR (NGO)) OR (INGO)) OR (community leader*)) OR ("community member")) OR (school)) OR (club)) OR (academia)) OR (researcher)) OR (committee) AND (english[Filter])) AND (community- based research) | 210286 |
| #4 | (((((((("developing country") OR ("low-income countries")) OR ("low and lower-middle-income countries")) OR ("low and lower-middle income countries",)) OR ("low and lower-middle income countries")) OR ("underdeveloped countries")) OR (third world)) OR (LMIC)) OR (LMICs) | 186207 |
| #3 | (((((((("community-based research") OR ("community-based participatory research")) OR (co-design)) OR (coproduction)) OR (cocreation)) OR (research)) OR (evaluation)) OR ("program plan*")) OR ("implementation research") | 16081949 |
| #2 | ((((((((((("maternal health") ) OR (newborn health)) OR (child health)) OR ("antenatal service")) OR (antenatal checkup*)) OR (institutional delivery)) OR (postnatal service*)) OR ("neonatal health")) OR ("prenatal care")) OR ("perinatal care")) OR ("maternal mental health") | 1076927 |
| #1 | ((((((((((((((patient) OR (mother*)) OR (consumer)) OR (client*)) OR (husband)) OR (pregnant mother*)) OR ("pregnant women")) OR ("mother-in-law")) OR (caregiver)) OR ("postpartum women")) OR ("lactating mother")) OR ("new mother")) OR (newborn)) OR (neonate)) OR (service user*) | 8623208 |
