# Supplementary material for: Understanding service users and other stakeholders’ engagement in maternal and newborn health services research: A systematic review of evidence from low- and middle-income countries
Source: PLoS One. 2024 Nov 27;19(11):e0309888. doi: 10.1371/journal.pone.0309888 (PMC11602069; doi:10.1371/journal.pone.0309888)
Supplement: S5 File — (DOCX) [file pone.0309888.s005.docx]

S3 File: Risk of bias/Quality assessment of included studies

1. Quality assessment of qualitative studies

| **Quality assessment of qualitative studies** | **Qualitative studies** | | | | |
| --- | --- | --- | --- | --- | --- |
| **Assessment criteria** | Baker et al. (2018) | Ekirapa-Kiracho et al. (2016) | George et al. (2018) | Mafuta et al. (2017) | Pallangyo et al. (2018) |
| **Qualitative study** |  |  |  |  |  |
| Is the qualitative approach appropriate to answer the research question? | Y | Y | Y | Y | Y |
| Are the qualitative data collection methods adequate to address the research question? | Y | Y | Y | Y | Y |
| Are the findings adequately derived from the data? | Y | Y | N | Y | Y |
| Is the interpretation of results sufficiently substantiated by data? | Y | Y | Y | Y | Y |
| Is there coherence between qualitative data sources, collection, analysis and interpretation? | Y | ? | ? | ? | Y |
| Yes=Y, No=N, Can't tell=? |  | | | | |

1. Quality Assessment of experimental studies

| **Quality assessment of experimental studies** | **Randomised controlled trials** | | | | | | | | | | **Non -Randomised controlled trails** | | | | | | |
| --- | --- | --- | --- | --- | --- | --- | --- | --- | --- | --- | --- | --- | --- | --- | --- | --- | --- |
| **Assessment criteria** | Goudar et al (2015) | Morrison et al (2020) | Manandhar et al (2004) | Tripathy et al (2016) | Amosse et al (2023) | Persson et al (2013) | Hoodbhoy et al (2021) | Alhassan et al (2019) | Azad et al (2010) | Fottrell et al (2016) | Dhital et al (2019) | Hossain and Ross (2006) | Ahluwalia et al (2003) | Thapa et al (2019) | Bich et al (2015) | Mwaniki et al (2014) | Hounton et al. (2009) |
| **Randomised controlled trials** |  |  |  |  |  |  |  |  |  |  |  |  |  |  |  |  |  |
| Is randomization appropriately performed? | Y | Y | Y | Y | Y | Y | Y | Y | Y | Y |  |  |  |  |  |  |  |
| Are the groups comparable at baseline? | Y | Y | Y | Y | Y | Y | N | Y | N | Y |  |  |  |  |  |  |  |
| Are there complete outcome data? | Y | Y | Y | Y | Y | Y | Y | Y | Y | Y |  |  |  |  |  |  |  |
| Are outcome assessors blinded to the intervention provided? | N | N | N | N | N | N | N | N | N | N |  |  |  |  |  |  |  |
| Did the participants adhere to the assigned intervention? | ? | ? | Y | Y | ? | ? | Y | ? | Y | ? |  |  |  |  |  |  |  |
| **Non -Randomised controlled Trails** |  |  |  |  |  |  |  |  |  |  |  |  |  |  |  |  |  |
| Are the participants representative of the target population? |  |  |  |  |  |  |  |  |  |  | Y | Y | ? | Y | Y | Y | ? |
| Are measurements appropriate regarding both the outcome and intervention (or exposure)? |  |  |  |  |  |  |  |  |  |  | Y | Y | Y | Y | Y | ? | Y |
| Are there complete outcome data? |  |  |  |  |  |  |  |  |  |  | Y | ? | ? | Y | Y | Y | ? |
| Are the confounders accounted for in the design and analysis? |  |  |  |  |  |  |  |  |  |  | ? | ? | ? | Y | ? | Y | ? |
| During the study period, is the intervention administered (or exposure occurred) as intended? |  |  |  |  |  |  |  |  |  |  | Y | ? | ? | ? | ? | Y | ? |
| Yes=Y, No=N, Can't tell=? |  | | | | | | | | | | | | | | | | |

1. Quality assessment of mixed method studies

| **Quality assessment of mixed methods studies** | **Mixed Method Studies** | | | |
| --- | --- | --- | --- | --- |
| **Assessment criteria** | Maluka et al (2023) | Datiko et al (2019) | Tancred et al (2014) | Kavi et al (2022) |
| Is there an adequate rationale for using a mixed methods design to address the research question? | Y | Y | Y | Y |
| Are the different components of the study effectively integrated to answer the research question? | Y | Y | Y | Y |
| Are the outputs of the integration of qualitative and quantitative components adequately interpreted? | ? | ? | ? | Y |
| Are divergences and inconsistencies between quantitative and qualitative results adequately addressed? | ? | Y | ? | N |
| Do the different components of the study adhere to the quality criteria of each tradition of the methods involved? | Y | Y | ? | ? |
| Yes=Y, No=N, Can't tell=? |  | | | |

Considering the quality assessment criteria of the MMAT tool, out of the total included studies, nine studies were considered high-quality, thirteen were considered medium-quality, and four were categorised as low-quality. Among qualitative studies, three studies were high quality, and two studies were medium quality; among experimental studies, five studies were high quality, nine studies were medium quality, and three were low quality; and among mixed method studies, one study was high quality, two studies were medium quality, and one study was low quality.
